# Perceived stress and its predictors in women with threatened preterm labour: A cross‐sectional study

**DOI:** 10.1002/nop2.1055

**Published:** 2021-09-22

**Authors:** Maryam Najjarzadeh, Shamsi Abbasalizadeh, Sakineh Mohammad‐Alizadeh‐Charandabi, Mohammad Asghari‐Jafarabadi, Mojgan Mirghafourvand

**Affiliations:** ^1^ Students’ Research Committee Faculty of Nursing and Midwifery Tabriz University of Medical Sciences Tabriz Iran; ^2^ Women's Reproductive Health Research Center Tabriz University of Medical Sciences Tabriz Iran; ^3^ Department of Midwifery Faculty of Nursing and Midwifery Social Determinants of Health Research Center Tabriz University of Medical Sciences Tabriz Iran; ^4^ Department of Statistics and Epidemiology Faculty of Health Road Traffic Injury Research Center Tabriz University of Medical Sciences Tabriz Iran; ^5^ Clinical Research Development Unit Imam Reza General Hospital Tabriz University of Medical Sciences Tabriz Iran

**Keywords:** prediction, pregnancy, preterm birth, psychological stress, women

## Abstract

**Aim:**

To determine prevalence and predictors of perceived stress in women with threatened preterm labour.

**Design:**

Cross‐sectional.

**Methods:**

We recruited 409 women with threatened preterm labour, hospitalized at two tertiary hospitals. We assessed their socio‐demographic and obstetrics characteristics, and their perceived stress, perceived social support, experience of violence using validated scales. Multiple linear regression was used to determine the predictors.

**Results:**

Data from all recruited women were analysed. Most of them had borderline (36%) or high (42%) level stress. Overall, 17 predictors were identified explaining 89.5% of variation in the stress score. Predictors of the higher stress score included: urban living, experience of sexual and psychological violence, perceived insufficient social support, experience of vaginal bleeding during current pregnancy, abnormal results in initial pregnancy tests, having multiple roles at home, being less than 28 weeks pregnant, being parous, sleep disorders, history of health problems, insufficient income and unwanted pregnancy.

## 1 INTRODUCTION

Threatened preterm labour is defined as regular uterine contractions in the absence of cervical change or ruptured membranes that is occurred after the gestation of viability and before 37 completed weeks of gestation (Hezelgrave & Shennan, 2017). It is the most common cause of hospitalization during pregnancy (Bacak et al., 2005). There are limited studies on incidence of threatened preterm labour. In a prospective cohort study in the United States, the incidence of first‐time hospitalization for threatened preterm labour was 9% and of whom 38% resulted in preterm birth in the first episode (McPheeters et al., 2005).

Threatened preterm labour could adversely affect women and their foetuses/infants. The recent meta‐analysis including 18 studies indicates high prevalence of psychological distress among women with antepartum hospitalizations for obstetric complications (depression 34%, 95% CI 21%–41% and anxiety 29%, 95% CI 16%–43%), which is about twice as prevalent as in the general obstetric population (Toscano et al., 2021). Also, mothers facing preterm delivery usually have different emotional reactions compared to those with full‐term birth; their stress and anxiety is the origin of psychological trauma, which can lead to post‐traumatic stress disorder and have adverse effect on the mother‐infant interaction (Eutrope et al., 2014). Preterm birth could also affect woman's future fertility because it can be associated with complications such as placental abruption, postpartum haemorrhage and even hysterectomy (Downes et al., 2017).

Preterm infants are at higher risk of mortality and adverse short‐ and long‐term outcomes (Crump, 2020; Dong & Yu, 2011). According to the global burden of disease study, complications from preterm birth rank eighth on the disability adjusted life years (DALYs) measurement (Murray et al., 2012). Studies also indicate that infants who are born at‐term and whose mothers experienced an episode of threatened preterm labour are at increased risk for foetal growth restriction (Campbell et al., 2012; Espinoza et al., 2007; Zoabi et al., 2013), and impaired cognitive development in childhood (Houben et al., 2019; Paules et al., 2017).

We found no study on prevalence of stress among women with threatened preterm labour. In our previous study in the study setting (Tabriz‐Iran) on the general pregnant women, prevalence of moderate‐to‐very severe stress were 15% in the second trimester and 21% in the third trimester (Iranzad et al., 2014). Stress during pregnancy is associated with strong and long‐term effects on the health of the woman and infant. It results in increasing level of cortisol and secretion of catecholamines, which in turn, could mediate factors affecting foetal development (Glover, 2014; Grote et al., 2010). Infants of mothers with high experience of psychological stress during pregnancy are more likely to have intrauterine growth retardation due to utero‐placental circulation disorders. They may also be born with a lower weight and low apgar score, have a smaller head circumference, and even poorer cognitive and language skills at older ages. The mothers may also develop eating and weight gain disorders and be more inclined to smoke and drink alcohol (Grote et al., 2010; Marcus, 2009).

Stress is one of the known factors that, through the hypothalamic‐pituitary‐adrenocortical (HPA) axis and its endocrine responses, could cause the activation of decidua and embryonic membranes, resulting in the secretion of prostaglandins and matrix metalloproteinases inducing premature cervical dilation and premature contractions (Rubens et al., 2014).

In modern medicine, predictors are used to estimate the probability of experiencing a specific health outcome. These risk factors, which are usually derived from regression models facilitate decision‐making related to health issues (Grant et al., 2018). Limited studies performed on the predictors of perceived stress during pregnancy indicate need for multifaceted approach to determine the predictors (Pais & Pai, 2018). However, we found no study examining predictors of stress in women with threatened preterm labour. Therefore, this study was performed to determine prevalence of perceived stress, and socio‐demographic, medical, obstetrics and psychological predictors of perceived stress score in women with threatened preterm labour.

## 2 THE STUDY

### 2.1 Design

This cross‐sectional study is part of a hospital‐based cohort study entitled "Risk factors and predictors of preterm birth in women with threatened preterm labour." The study was performed in Alzahra and Taleghani teaching hospitals (the only tertiary level hospitals offering specialized care for premature infants in the city of Tabriz, the capital of East Azarbaijan province) on women hospitalized with symptoms of preterm labour. Alzahra hospital offers 7/24 premature neonatal care, that is, its specialized premature neonatal care (such as the neonatal intensive care services, neonatal intensive care unit (NICU) services and presence of a neonatal specialist) is given 7 d/week and 24 hr/d, with 41 NICU active beds. Taleghani is also a tertiary level hospital in terms of neonatal services, but its specialized care in terms of presence of a neonatologist at hospital is limited to non‐holiday morning shifts, and it has 24 NICU active beds. All women less than 32 weeks pregnant threatened with preterm labour were referred from East Azarbaijan province and in some cases from neighbouring provinces to Alzahra center. Thus, the number of pregnant women admitted to Alzahra hospital due to the threat of preterm labour was much higher than Taleghani hospital.

### 2.2 Participants and procedure

The study population consisted of 24^+0^–36^+4^ weeks pregnant women who had healthy foetuses and were hospitalized for threatened preterm labour, that is, had regular uterine contractions (at least one every 10 min) in the absence of cervical change or ruptured membranes. Exclusion criteria were: inability to read and/or write in Persian, more than triplet pregnancies, no Iranian nationality (themselves or their spouses).

To complete the data, the first author (MN) conducted face‐to‐face interviews with the participants in a private and quiet environment in the high‐risk pregnancy or in birth wards, in the first 24 hr of admission (after her stabilization in the inpatient ward) during the morning or evening shifts on non‐holidays. Each interview lasted from 35 to 45 min, according to the participant's clinical condition. To counteract the social acceptance bias (Gray et al., 2017), we used coded anonymous questionnaires and assured all the women before the interview about confidentiality of their information, also sensitive questions such as intimate violence were placed at the end of the questionnaires.

In order to calculate the sample size in predictive studies using a regression model with six or more predictors, at least 10 participants are required for each predictor. However, it is recommended that to obtain ideal results, to enhance the study power, and to detect small effect sizes, 30 samples per a predictor factor should be included in the study (Voorhis & Morgan, 2007); hence, we included 409 pregnant women in this study. Considering the identification 6, 15, and 17 predictors for the three models under study, this number of samples is sufficient for the first model in ideal condition and is sufficient for the second and third models (respectively 27 and 24 samples for each predictor) in completely acceptable conditions.

### 2.3 Data collection

Data collection tools included a questionnaire about demographic, social, medical and obstetrics characteristics, the Cohen's perceived stress scale (PSS‐10) (Cohen et al., 1983), Zimet's multidimensional scale of perceived social support (MSPSS‐12) (Zimet et al., 1988), and the WHO violence against women (VAW‐13) (García‐Moreno et al., 2005). The content and face validity of the questionnaire on demographic and social and medical and obstetrics characteristics, which were developed by reviewing the literature, were determined by 10 experts from the Tabriz University of Medical Sciences.

Perceived Stress Scale (PSS‐10): It is a 10‐item five‐point Likert scale with the options “never” to “very much” (scored zero–four), which examines perceived stress over the past month. The range of stress scores that are obtained from sum scores of the items is between zero and 40, and the higher the score, the greater the perceived stress. The sum scores 13 and lower indicates normal stress, 14–19 reveals borderline stress, and 20 and higher indicates high stress requiring treatment (Cohen et al., 1983).

Multidimensional Scale of Perceived Social Support (MSPSS): It is a 12‐item, five or seven‐point Likert scale. We used the five‐point Likert scale (from strongly disagree: one point to strongly agree: five points). The overall score range was obtained from the average scores of the items (Zimet et al., 1988). The scores indicate: 1.0–2.33 low support, 2.34–3.67 moderate support and 3.68–5.0 high support (Zimet, 2016).

The validity and reliability of the PSS (Khalili et al., 2017) and MSPSS (Bagherian‐Sararoudi et al., 2013) have already been confirmed in Iranian society by psychometric studies. In the present study, the reliability of the scales was evaluated by test retest at intervals of two weeks on 20 subjects, also by determining the internal consistency. Reliability of both scales using Cronbach's α and intra‐class correlation coefficient (ICC) were acceptable; that is, perceived stress: Cronbach's α 0.72, ICC 0.72 (95% CI 0.65–0.85) and perceived social support: Cronbach's α 0.82, ICC 0.82 (95% CI 0.75–0.90).

WHO’s Violence against Women (WHO‐VAW): The scale has 13 items with four options (never, once, sometimes and often) and evaluates violence in three dimensions: psychological (four items), physical (six items) and sexual (three items). This scale was used in WHO multicentre studies in 10 countries in 2005 (García‐Moreno et al., 2005), and its validity and reliability have been confirmed in Brazil among pregnant women (Ribeiro et al., 2014; Schraiber et al., 2010) and in Sweden (Nybergh et al., 2013) by psychometric methods. We used this questionnaire in the current study after obtaining written permission (by e‐mail) from WHO and after the translation and back translation process, determining the content validity ratio (CVR), content validity index (CVI), item impact and determining its reliability using test re‐test on 20 pregnant women and internal consistency. In total and in the three dimensions, Cronbach's α was 0.7–0.8 and ICC was 0.80–0.82. Details of the psychometric results of this scale will be published in another report. To determine the existence of experienced violence, as in the WHO multicentre study (Nybergh et al., 2013; Schraiber et al., 2010), we considered it as a binary variable, that is, sum score of one or higher in each dimension was considered as experience of violence at that dimension and in case of experience of violence in at least one dimension, the overall experience of violence was considered positive.

Sleep quality satisfaction was assessed using one four‐point Likert question with options of “not at all, a little, average and high”. In determining the risk factors, the option “not at all” was considered as dissatisfaction with sleep quality.

### 2.4 Data analysis

Normality of the distribution of the perceived stress score was confirmed by examining skewness and kurtosis. In the first step, using the unadjusted general linear model, we examined the relationship between each probable predictive variable with perceived stress score. Then, to determine predictors of the perceived stress score, all of the variables with *p* < .2 in the unadjusted models were entered into multiple linear models (Agresti, 2012), using the backward strategy. Sidak was used for adjustment of the multiple comparisons. Before using regression models, the establishment of linear regression assumptions such as residual normality and absence of multicollinearity were examined. Variance inflation factor (VIF) less than five was considered as a sign of lack of seriousness multicollinearity between independent variables and no modelling problem. In each model, to determine what proportion of the variances of the dependent variable can be explained by the independent variables, we used adjusted R2. Data were analysed using the statistical package for the social sciences (SPSS), version 21 (Chicago, IL, USA) and *p*‐value levels less than .05 were considered statistically significant.

### 2.5 Ethics

This study was approved by the ethics committee of Tabriz University of Medical Sciences on May 2019 with the number IR.TBZMED.REC.1398.214. We obtained informed written consent from all participants before their recruitment. We designed and conducted this study in accordance with the Helsinki Declaration.

## 3 RESULTS

**FIGURE 1:**
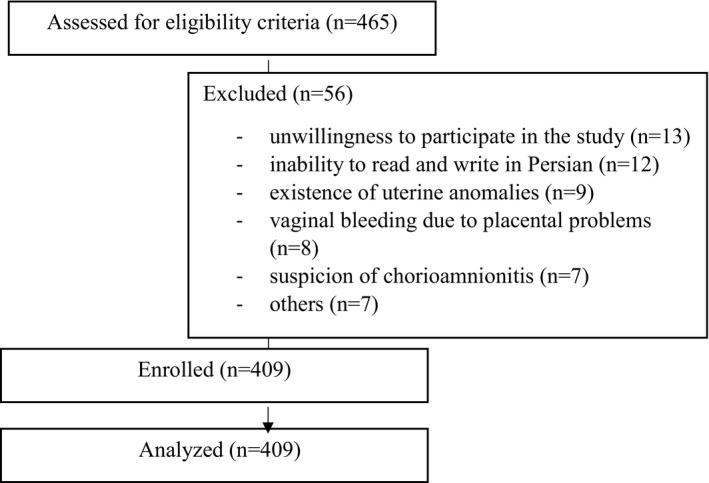
Study flow chart

From July 2019–August 2020 (during 14 months), 409 out of 465 hospitalized women with threatened preterm labour who were approached were included in the study and analysed (Figure 1). There was no missing value in the main questions of the included cases, except in three cases, which had 1–4 missing values in items of the VAW or MSPSS questionnaires. They were replaced by the series mean before further analysis.

Ninety‐four per cent of samples were collected from Alzahra hospital. Mean (*SD*) age of the women was 28.2 (6.7), and their own education was 11.0 (3.7) and their husbands’ education was 11.2 (3.8) year. Fifty‐two per cent were nulliparous, 73% were urban living and 82% were housewives. Eighty per cent reported sufficient or relatively sufficient income for living expenses, and about half of them (47%) had their own homes. All women were married, and 83% lived only with their spouse or spouse and child/children, and 7% cared for one or more elder people at home. The mean gestational age was 31.7 weeks [*SD* 3.0, range 24.0–36.2], 71 women (15.3%) had vaginal bleeding or spotting at the time of admission to the hospital. Most of the women (80%) had received two doses of corticosteroids to promote function of neonatal respiration system and 57% had received at least one tocolytic agent.

The mean perceived stress score was 18.7 [*SD*: 6.5, range: 3–39] and 35.9% had a borderline and 41.8% a high level stress score.

Among the socio‐demographic variables, five variables in the unadjusted analysis were related to perceived stress score with *p*‐value less than .05: urban living, insufficient household income, living with other family members (in addition to spouse and children), exposure to noise pollution at work or home and caring for one or more elder people at home (Table S1). These variables in addition to two variables of hard work (long working hours of more than 8 hr/d or obligation to standing and sitting for more than 45 min continuously at work) and exposure to second‐hand smoke (at work or home), which were related to the stress scores with *p* < .2 (seven factors) were entered into the first regression model and all of them, except the hard work, were identified as predictors of the stress score. The proportion of variation in the score explained by these independent variables was 79% (Table 1).

**TABLE 1:** Socio‐demographic predictors of perceived stress^†^ in women threatened with preterm labour (*N* = 409)

Predictors^‡^	*N*	Beta	B (95% CI)	*p*
Living in urban area	300	0.641	15.0 (14.0 to 16.0)	<.001
Living with others in addition to spouse and children	71	0.146	7.0 (4.5 to 9.5)	<.001
Insufficient household income	83	0.124	5.5 (3.5 to 7.5)	<.001
Being passive smoker^§^	137	0.126	4.5 (2.5 to 6.0)	<.001
Exposure to noise pollution at work or home	94	0.102	4.0 (2.0 to 6.5)	<.001
Caring for elder person/s at home	29	0.069	5.0 (1.5 to 9.0)	.009

Note

All analysis were done using the multiple linear regression model with the backward strategy. Sidak was used to adjust for the multiple comparisons. There was no high multicollinearity between the independent variables in the model (VIFs <1.5).

^†^Measured by perceived stress scale (PSS‐10) with attainable range score 0–40; the higher score, the more stress.

^‡^Adjusted for all demographic and socio‐economic variables with a relation of *p* < .2 in the unadjusted analyses, excluded variable: Long working hours, adjusted *R* ^2^ = 0.793,

^§^Self‐reported exposure to environmental, second‐hand tobacco smoke (cigarette or hookah).

Among the medical, obstetrics and psychological variables, 15 variables were related to the perceived stress score with *p*‐value less than .05 in the unadjusted analysis: a history of stillbirth and miscarriage, self‐referral to the hospital for current hospitalization, history of health problems before the current pregnancy, being less than 28 weeks pregnant, abnormal results in the initial pregnancy tests, perceived low or moderate social support, experience of psychological, physical, or sexual violence during the current pregnancy, history of vaginal bleeding during the current pregnancy, average sleep less or more than 8–9 hr during the day or night, dissatisfaction with sleep quality during the past month, intention to induce abortion in the current pregnancy and the history of hospitalization during the current pregnancy (Table S2). These variables in addition to two variables of being parous and unwanted pregnancy, which were related to the stress scores with *p* < .2 were entered in the second regression model. All of the variables, except the intention to induce abortion in the current pregnancy and history of stillbirth, remained in the model and were detected as stress score predictors. The proportion of variation in the stress score explained by these independent variables was 87% (Table 2). The variable of experience of any type of violence, which has a statistically significant relation with the stress score (*p* < .001) in the unadjusted analysis (Table S2) was entered in none of the regression models, because of high multicollinearity with other independent variables.

**TABLE 2:** Medical, obstetrics and psychological predictors of perceived stress^†^ in women threatened with preterm labour (*N* = 409)

Predictors^‡^	*N*	Beta	B (95% CI)	*p*
Experience of psychological violence during the current pregnancy^§^	254	0.295	7.5 (6.0 to 9.0)	<.001
Perceived low or moderate social support^¶^ (Ref: high)	183	0.125	4.0 (2.5 to 5.0)	<.001
Self‐referred to hospital	175	0.122	4.0 (2.5 to 5.0)	<.001
History of vaginal bleeding during the current pregnancy	167	0.110	3.5 (2.0 to 5.0)	<.001
Abnormal results in the initial pregnancy tests^£^	175	0.101	3.0 (1.5 to 4.5)	<.001
Being parous	194	0.092	2.5 (1.0 to 4.0)	<.001
Being less than 28 weeks pregnant	48	0.072	4.0 (2.0 to 6.0)	<.001
Dissatisfaction with sleep quality during the past month	60	0.072	4.0 (2.0 to 6.0)	<.001
History of health problems before the current pregnancy^¥^	154	0.073	2.5 (1.0 to 4.0)	.001
History of hospitalization during the current pregnancy	69	0.063	3.0 (1.0 to 5.0)	.002
History of miscarriage	118	0.064	2.5 (1.0 to 4.0)	.003
Experience of sexual violence during the current pregnancy	53	0.056	3.0 (1.0 to 5.0)	.006
Average sleep less or more than 8–9 hr during the day or night	272	0.058	2.0 (0.5 to 3.5)	.007
Unwanted pregnancy	132	0.056	2.0 (0.5 to 3.5)	.011
Experience of physical violence during the current pregnancy	81	0.047	2.0 (0.5 to 3.5)	.030

Note

All analysis were done using the multiple linear regression model with the backward strategy. Sidak was used to adjust for the multiple comparisons. After excluding variable of “any type of violence,” there was no high multicollinearity between the independent variables in the model (VIFs <2.6).

^†^Measured by perceived stress scale (PSS‐10) with attainable range score 0–40; the higher score, the more stress.

^‡^Adjusted for all obstetrical, clinical and psychological variables with a relation of *p* < .2 in the unadjusted analyses, excluded variables: intention to induce abortion, history of stillbirth, adjusted *R* ^2^ = 0.871.

^§^Measured by WHO violence against women (VAW‐13), experience was considered as “yes” when women have marked once or sometimes or often for at least one of the relevant items

^¶^Measured by multidimensional scale of perceived social support (MSPSS‐12) with a range score of 1–5, 1.0–2.33 low support, 2.34–3.67 moderate support, 3.68–5.0 high support.

^£^Those who had at least one abnormal result in their initial pregnancy laboratory tests such as TSH (Thyroid Stimulating Hormone), FBS (Fast Blood Sugar), CBC (Complete Blood Count), urine analysis and others.

^¥^Including diabetes mellitus, hypertension, hypo/hyperthyroid, anaemia, renal or cardiovascular diseases, infertility and others.

By entering all the factors included in the previous two models into the third model (24 variables), 17 predictors of perceived stress in women threatened with preterm labour were extracted as follows: urban living (β = 0.254), experience of psychological violence (β = 0.240), perceived low or moderate social support (β = 0.102), history of vaginal bleeding during the current pregnancy (β = 0.091), abnormal results in the initial pregnancy tests (β = 0.082), self‐referred to hospital during the current hospitalization (β = 0.073), living with other family members (in addition to spouse and children) (β = 0.071), being less than 28 weeks pregnant (β = 0.066), history of hospitalization during the current pregnancy (β = 0.051), being parous (β = 0.064), history of health problems before the current pregnancy (β = 0.049), dissatisfaction with sleep quality during the past month (β = 0.047), unwanted pregnancy (β = 0.044), experience of sexual violence (β = 0.044), insufficient household income (β = 0.042), caring for one or more elder people at home (β = 0.042) and exposure to noise pollution at their workplace or home (β = 0.041). The proportion of variation in the stress score explained by these independent variables was 89.5% (Table 3).

**TABLE 3:** Overall predictors of perceived stress† in women threatened with preterm labour (*N* = 409)

Predictors^‡^	*N*	Beta	B (95% CI)	*p*
Living in urban area	300	0.254	6.0 (4.5 to 7.0)	<.001
Experience of psychological violence during the current pregnancy^§^	254	0.240	6.0 (5.0 to 7.0)	<.001
Perceived low or moderate social support^¶^ (Ref: high)	183	0.102	3.0 (2.0 to 4.0)	<.001
History of vaginal bleeding during the current pregnancy	167	0.091	3.0 (1.5 to 4.0)	<.001
Abnormal results in the initial pregnancy tests^£^	175	0.082	2.5 (1.0 to 4.0)	<.001
Living with others in addition to spouse and children	71	0.071	3.5 (1.5 to 5.0)	<.001
Being less than 28 weeks pregnant	48	0.066	4.0 (2.0 to 6.0)	<.001
Self‐referred to hospital	175	0.073	2.0 (1.0 to 3.5)	.001
History of hospitalization during the current pregnancy	69	0.051	2.5 (1.0 to 4.0)	.006
Being parous	194	0.064	2.0 (0.5 to 3.0)	.007
Dissatisfaction with sleep quality during the past month	60	0.047	2.5 (0.5 to 4.0)	.009
Experience of sexual violence during the current pregnancy	53	0.044	2.5 (0.5 to 4.5)	.015
History of health problems before the current pregnancy^¥^	154	0.049	1.5 (0.5 to 3.0)	.020
Insufficient household income	83	0.042	2.0 (0.5 to 3.5)	.026
Caring for elder person/s at home	29	0.042	3.0 (0.5 to 6.0)	.027
Unwanted pregnancy	132	0.044	1.5 (0.1 to 3.0)	.036
Exposure to noise pollution at work or home	94	0.041	2.0 (0.1 to 3.5)	.042

Note

All analysis were done using the multiple linear regression model with the backward strategy. Sidak was used to adjust for the multiple comparisons. After excluding variable of “any type of violence,” there was no high multicollinearity between the independent variables in the model (VIFs <3.1).

^†^Measured by perceived stress scale (PSS‐10) with attainable range score 0–40; the higher score, the more stress.

^‡^Adjusted for all socio‐demographic, obstetrical, clinical and psychological variables with a relation of *p* < .2 in the unadjusted analyses, excluded variables: being passive smoker, long working hours, history of miscarriage, history of stillbirth, average sleep less or more than 8–9 hr during the day or night, experience of physical violence during the current pregnancy, intention to induce abortion., adjusted *R* ^2^ = 0.895.

^§^Measured by WHO violence against women (VAW‐13), experience was considered as “yes” when women have marked once or sometimes or often for at least one of the relevant items

^¶^Measured by multidimensional scale of perceived social support (MSPSS‐12) with a range score of 1–5, 1.0–2.33 low support, 2.34–3.67 moderate support, 3.68–5.0 high support.

^£^Those who had at least one abnormal result in their initial pregnancy laboratory tests such as TSH (Thyroid Stimulating Hormone), FBS (Fast Blood Sugar), CBC (Complete Blood Count), urine analysis and others.

^¥^Including diabetes mellitus, hypertension, hypo/hyperthyroid, anaemia, renal or cardiovascular diseases, infertility and others.

## 4 DISCUSSION

Mean perceived stress score of the hospitalized women with threatened preterm labour was relatively high (46.7, *SD* 16.2 from attainable score of 0–100). The majority of them had high (41.8%) or borderline (35.9%) stress levels. Urban living, experience of psychological or sexual violence, perceived low or moderate social support, history of vaginal bleeding during the current pregnancy, abnormal results in the initial pregnancy tests, living with other family members (in addition to spouse and children), being less than 28 weeks pregnant, self‐referral to hospital for current hospitalized, history of hospitalization during the current pregnancy, being parous, dissatisfaction with sleep quality during the past month, unwanted pregnancy, history of health problems before the current pregnancy, inadequate household income, caring for one or more elder people at home, and exposure to noise pollution at work or home were predictive factors explaining a high proportion of variation (89.5%) of the perceived stress score in women threatened with preterm labour.

We found no study examining perceived stress in women at risk of preterm birth in Iran or other countries. The stress mean score in our study was remarkably higher than the score of pregnant women referred to health centres in two studies conducted in the same city (Tabriz). The mean stress score in one of the previous studies using the same scale was 28.7 (Iranzad et al., 2014), and in the other study using the depression, anxiety and stress scale (DASS‐21) was 30.2 (Effati‐Daryani et al., 2018) from attainable range score of 0–100. Also in this study, the frequency of women with high stress was higher than that in our previous study (42% versus. 12%) (Effati‐Daryani et al., 2018). These results may indicate remarkably higher levels of stress in hospitalized women with threatened preterm labour than in other pregnant women. High stress in these women may be related to the higher risk of preterm labour in the women with high stress. Another possible reason for this high stress could be related to neonatal problems of the premature infants, as the present study also indicated that the stress score of women less than 28 weeks pregnant was significantly higher than that of women who were 28–36 weeks pregnant. Other reasons, such as the lower frequency of high perceived social support and the higher frequency of individuals with enduring illness in the present study compared to the previous studies may also have been contributed in the higher stress score. However, based on the results of this cross‐sectional study, without having a control group, it is not possible to comment on the higher perceived stress of hospitalized women threatened with preterm labour compared to other pregnant women.

Syndemic theory is a biosocial theory that is widely recognized in various fields of public health such as the prevention and treatment of diseases, sexual and reproductive health, nursing, medicine and psychology (Program Collaboration and Service Integration). According to this theory, all forms of deviance from human health are likely to be developed and exacerbated under conditions of social inequalities such as poverty, stigmatization and structural violence, and co‐occurrence of multiple social problems and illness or deviation from a good health status can further threaten the condition of the affected person (Singer et al., 2017). Therefore, according to the syndemic theory, high stress in hospitalized pregnant women with symptoms of preterm labour, especially in women with other co‐morbidities such as prepregnancy health problems, history of abnormal results in the initial pregnancy tests, history of vaginal bleeding during the current pregnancy, or existence of socio‐economic problems such as insufficient household income, playing multiple roles in the family (spouse, mother, caregiver, daughter‐in‐law) can be justified.

The results of the present study on urban living (the strongest factor), exposure to noise pollution at work or home, and insufficient household income as predictors of perceived stress are consistent with the body of knowledge that has examined the relationship between stress and urban living. Studies have found the role of nature and natural green spaces in reducing psychological stress and cortisol (Ewert & Chang, 2018), while urban living often harms individual mental health and increases their stress with alienation of human beings from nature, air and noise pollution, crowding and congestion, inequality, and even violent behaviours (Dekker et al., 2008; Gruebner et al., 2017; Morozov, 2018; Srivastava, 2009).

The results of the present study on the experience of psychological and sexual violence as predictors of high perceived stress are consistent with the results of other studies (Ellsberg et al., 2008; Fisher et al., 2012; Kashanian et al., 2019). Violence is one of the most important social determinants of health (Oram et al., 2019). Perceiving and experiencing violence acts as a trigger for the biological stress system; and with releasing hormones such as cortisol, epinephrine, corticotropin‐releasing hormone (CRH), dehydroepiandrosterone (DHEA) and neuropeptides exposes human's body to physical and mental complications and could result in psychological stress (Black, 2011).

Receiving less social support during pregnancy as a strong predictor of perceived stress is also in line with the results of a systematic review (Fisher et al., 2012) and our previous study in Tabriz (Iranzad et al., 2014) and shows the importance of providing adequate social support during pregnancy. Non‐human (Wittig et al., 2016) and humans models (Ditzen & Heinrichs, 2014) that have studied the effect of social support on stress have shown that receiving supportive behaviours from others helps to regulate hypothalamic‐pituitary‐adrenocortical (HPA) axis and reduce cortisol secreted by the adrenal glands, and so leading to stress reduction (Ditzen & Heinrichs, 2014; Wittig et al., 2016). Studies in adults have shown that people who do not receive adequate support from their social networks (family, friends and others) experience a disruption in the structure (number and frequency of social connections) and function (social support) of their social support networks and have loneliness and isolation feelings (Menec et al., 2020). These feelings are associated with physical and mental health consequences such as brain dysfunction, sleep disturbance, cortisol secretion, deficiency in cellular and humoral immunity and decreased inflammatory responses, and these individuals report more psychological distress (Leigh‐Hunt et al., 2017; Menec et al., 2020).

The results of our study on caring for one or more elder people at home and living with other family members (in addition to one spouse and children, who in Iranian society often include the father‐in‐law, mother‐in‐law and other members of the spouse's family) as predictors of perceived stress are consistent with a study conducted in the United States showing an increase in cortisol and perceived stress in caregivers of the one or more elder people at home (Gallagher‐Thompson et al., 2006). This high stress may be due to difficulty of playing multiple roles in the family and community (Stewart et al., 2019; Sumra & Schillaci, 2015), or due to individual dissatisfaction with these roles (Sumra & Schillaci, 2015). On the other hand, some believe that multiple roles in men and women improve the quality of their relationships, give them a sense of usefulness and purpose, and create more positive emotions in them (Ahrens & Ryff, 2006). These contradictory results reveal the need for further studies in this area.

Sleep disorders as a predictor of high perceived stress are in line with the results of a study in Riyadh, Saudi Arabia on pregnant women (Ahmed et al., 2017). It has been shown that following sleep disorders in adults, the secretion of catecholamines and the adrenocorticotropin hormone, and finally serum cortisol increases, which leads to occurrence of the symptoms of physiological and psychological stress (Medic et al., 2017).

In our study, being parous and unwanted pregnancy were identified as predictors of perceived stress. There was no relationship in the Saudi study (Ahmed et al., 2017) between number of children and perceived stress and in a study in Tehran‐Iran (Kashanian et al., 2019) between unwanted pregnancy and perceived stress of pregnant women. However, in the study of pregnant Pakistani women, unwanted pregnancy and having more children were identified as predictors of stress (Waqas et al., 2020). Secondary analysis of a prospective cohort study in the United States has also shown that unwanted pregnancies was associated with higher perceived stress in pregnant women (Gariepy et al., 2016). A large study in Finland found that parous women were 1.9 times more fearful of giving birth than nulliparous (Räisänen et al., 2014). There is a relationship between fear of childbirth and psychological disorders such as anxiety and post‐traumatic stress disorder (Rouhe et al., 2011). In parous women, the fear of childbirth is often due to their traumatic previous childbirth and negative childbirth experiences (Hofberg & Ward, 2003). The study of Ghanbari et al. in the same setting of our study has indicated the high prevalence of negative childbirth experiences (Ghanbari‐Homayi et al., 2019).

Being less than 28 weeks pregnant, which in case of childbirth would result in a very premature infant, was shown to be a predictor of high perceived stress in women. Since most premature infants require specialized care and if they survive, some of them will suffer from long‐term neurological complications (Blencowe et al., 2013), very preterm birth imposes a great deal of psychological burden on parents. The results of our study are consistent with previous studies (Eutrope et al., 2014; Sawyer et al., 2013).

Consistent with the results of the present study, a study in Tehran‐Iran has also shown that previous hospitalization (for foetal or maternal complications) increases the perceived stress of pregnant women (Kashanian et al., 2019). Hospitalization, whether due to complications threatening the continuation of pregnancy, such as the threat of miscarriage and vaginal bleeding (Semczuk et al., 2004) or due to underlying diseases of the pregnant woman and pregnancy complications can be a risk factor for high perceived stress in pregnant women (Ahmed et al., 2017). Also, the results of the present study on the existence of chronic diseases and health problems before the current pregnancy are in line with the results of a study in Riyadh‐Saudi Arabia (Ahmed et al., 2017). Moreover, the results of a study done in Tehran‐Iran about previous vaginal bleeding during the current pregnancy as a predictor of perceived stress are similar to the current study (Kashanian et al., 2019).

Contrary to our expectations, the results of the present study showed that women who self‐referred to the hospitals had higher perceived stress scores than women who either were referred or dispatched to the hospitals. We found no explanation for such a result. Also, no original study was found that compared the two groups. However, a study based on the case reports in the United Kingdom report that referral and dispatching of pregnant women to more well‐equipped centres imposes additional psychological stress on them (Musson & Harrison, 2016; Watson et al., 2020). This report does not appear to be consistent with the results of our study. Therefore, further studies in this field seem necessary to determine the probable reason.

Overall, the results of this study confirm multifaceted and biosocial nature of psychological stress and emphasize on a holistic approach to prevention, treatment and policies related to this public health problem. Midwives and other healthcare workers who care for pregnant women need to do early screening and timely interventions to reduce the preventable factors identified in this study, including providing social support, reducing domestic violence and improving sleep quality, to reduce the stress of the pregnant women and maternal‐foetal complications of high perceived stress.

### 4.1 Limitations

The relatively high number of samples, which made it possible to determine a large number of predictors in almost ideal conditions and the high goodness of the fit measure of the models can be considered as the positive points of this study. Completing the perceived stress questionnaire in the first hours of hospital admission (after stabilizing the patient in the ward) prevented influencing the atmosphere of the hospital on the pregnant women's responses. Collecting data in the same way by one person (the first author) and by face‐to‐face interviews minimized the opportunity of non‐response bias. Another strength of our research environment was that we were able to cover the majority of hospitalized women threatened with preterm labour (and almost all women under 32 weeks of pregnancy) in the province and neighbouring provinces. Therefore, the subjects had a high diversity and this increased generalizability of the results.

Due to the nature of cross‐sectional studies, the relationships indicated in this study cannot be considered as a cause‐effect relationship. Therefore, conducting studies providing higher levels of evidence, including clinical trials are recommended to determine the effect of some controllable factors such as interventions to prevent intimate partner violence and promote social support in these women on their stress levels. Also cohort studies could help to determine the direction of some of the relationships identified in this study. Case‐control studies can also be helpful to compare the stress of pregnant women with the threatened preterm labour with the stress of other pregnant women.

## 5 CONCLUSIONS

The prevalence of perceived stress in hospitalized women threatened with preterm labour is high. Urban living, experience of psychological and sexual violence, perceived low or moderate social support are the strongest predictors of the stress score. Other predictors include noise pollution at work or home, and dissatisfaction with sleep quality. Early screening and timely interventions for reducing the identified preventable factors (including providing social support, preventing domestic violence, reducing noise pollution at work or home, and improving sleep quality) may reduce the stress of the women.

## CONFLICT OF INTEREST

The authors have no conflicts of interest to declare.

## AUTHOR CONTRIBUTIONS

MN: Main idea, principal investigator of the study, literature search, data collection and data analysis and first draft write up, rewriting of the manuscript. ShAA: Co‐investigator of the study, field supervision, Manuscript editing. SMAC: Main idea, principal investigator of the study, literature search, data analysis, rewriting of the manuscript. MAJ: Co‐investigator of the study, data analysis, rewriting of the manuscript. MM: Main idea, Co‐investigator of the study, Manuscript editing. All authors approved the final version of the manuscript and agreement to be accountable for all aspects of the work.

## Supporting information

Supplementary Material

## Data Availability

The data sets used and analysed in this study can be made available by the corresponding author at reasonable request.
