# Supplementary material for: Perceived stress and its predictors in women with threatened preterm labour: A cross‐sectional study
Source: Nurs Open. 2021 Sep 22;9(1):210–21. doi: 10.1002/nop2.1055 (PMC8685874; doi:10.1002/nop2.1055)
Supplement: Supplementary file 1 — Supplementary Material [file NOP2-9-210-s001.docx]

**Table s1. Perceived stress^†^ score by socio-demographic factors (n=409)**

| Factors^‡^ | n | Mean (SD) | MD (95% CI) |
| --- | --- | --- | --- |
| Age (years) |  |  |  |
| < 20 | 43 | 18.0 (6.0) | reference |
| 20-35 | 295 | 18.5 (6.0) | 0.5 (-2.5 to 3.5) |
| ≥ 36 | 71 | 18.5 (6.5) | 0.5 (-2.0 to 3.0) |
| Woman education |  |  |  |
| Primary | 70 | 19.5 (7.0) | 1.0 (-1.0 to 3.0) |
| Secondary/diploma | 235 | 18.5 (6.0) | reference |
| Academic | 104 | 19.0 (6.0) | 0.5 (-1.5 to 2.5) |
| Age difference with husband (years) |  |  |  |
| Below husband | 37 | 18.0 (6.0) | reference |
| 0-5 | 181 | 19.0 (6.5) | 1.0 (-2.0 to 4.0) |
| ≥ 6 | 191 | 19.0 (6.5) | 1.0 (-2.0 to 4.0) |
| Husband education |  |  |  |
| Primary | 65 | 19.5 (6.5) | 1.0 (-1.0 to 3.0) |
| Secondary/diploma | 235 | 18.5 (6.5) | reference |
| Academic | 109 | 19.0 (6.0) | 0.5 (-1.5 to 2.5) |
| Household income |  |  |  |
| Sufficient/ partly sufficient | 326 | 18.0 (6.5) | reference |
| Insufficient | 83 | 20.0 (5.5) | **2.0 (0.5 to 3.5)^*^** |
| Long working hours^§^ |  |  |  |
| No | 25 | 17.0 (7.5) | reference |
| Yes | 48 | 20.0 (6.0) | 3.0 (-0.1 to 6.0) |
| Exposure to noise pollution at work or home |  |  |  |
| No | 315 | 18.0 (6.3) | reference |
| Yes | 94 | 21.0 (6.5) | **3.0 (1.5 to 4.5)^***^** |
| Living with others (in addition to spouse and children) |  |  |  |
| No | 338 | 18.5 (6.5) | reference |
| Yes | 71 | 20.0 (7.0) | **1.5 (0.1 to 3.0)^*^** |
| Caring for elderly person/s at home |  |  |  |
| No | 380 | 18.5 (6.5) | reference |
| Yes | 29 | 23.0 (6.3) | **4.5 (2.0 to 7.0)^***^** |
| Having under 5 years child at home |  |  |  |
| No | 333 | 18.5 (6.5) | reference |
| Yes | 76 | 19.0 (6.0) | 0.5 (-1.0 to 2.0) |
| Living location |  |  |  |
| Rural | 109 | 17.5 (6.5) | reference |
| Urban | 300 | 19.0 (6.5) | **1.5 (0.1 to 3.0)^*^** |
| Being passive smoker^¶^ |  |  |  |
| No | 272 | 18.5 (6.5) | reference |
| Yes | 137 | 19.5 (6.0) | 1.0 (-0.1 to 2.5) |

* P < 0.05, ** P < 0.005, *** P < 0.001,

† Measured by perceived stress scale (PSS-10) with attainable range score 0-40; the higher score, the more stress.

‡ all analysis were done using the univariate general linear model.

§ Long working hours (more than 8 hours in a day) or longtime standing or sitting on the job (more than 45 minutes continuously), 336 were housewives.

¶ Self-reported exposure to environmental, second-hand tobacco smoke (cigarette or hookah).

**Table s2. Perceived stress^†^ score by obstetrical, clinical and psychological factors (n=409)**

| **Factors^‡^** | **n** | **Mean (SD)** | **MD (95% CI)** |
| --- | --- | --- | --- |
| Being parous | | | |
| No | 215 | 18.5 (6.0) | reference |
| Yes | 194 | 19.5 (6.5) | 1.0 (-0.1 to 2.5) |
| History of vaginal bleeding during the current pregnancy | | | |
| No | 242 | 18.0 (6.0) | reference |
| yes | 167 | 20.0 (6.5) | **2.0 (0.5 to 3.5)^**^** |
| History of stillbirth | | | |
| No | 405 | 18.2 (6.5) | reference |
| Yes | 4 | 32.0 (7.2) | **13.8 (7.4 to 20.3) ^***^** |
| Preterm rupture of membranes | | | |
| No | 338 | 18.5 (6.5) | reference |
| Yes | 63 | 19.5 (6.0) | 1.0 (-1.0 to 3.0) |
| Doubtful | 8 | 22.0 (7.0) | 3.5 (-2.0 to 9.0) |
| History of miscarriage | | | |
| No | 291 | 18.0 (6.5) | reference |
| Yes | 118 | 20.0 (6.0) | **2.0 (0.5 to 3.0)^*^** |
| Self-referred to hospital | | | |
| No | 234 | 18.0 (6.0) | reference |
| Yes | 175 | 20.0 (7.0) | **2.0 (0.5 to 3.5)^*^** |
| Unwanted pregnancy | | | |
| No | 277 | 18.5 (6.0) | reference |
| Yes | 132 | 19.5 (7.0) | 1.0 (-0.2 to 2.5) |
| Multiple pregnancy | | | |
| No | 371 | 18.0 (7.0) | reference |
| Yes | 38 | 19.0 (6.5) | 1.0 (-1.5 to 3.0) |
| History of health problems before the current pregnancy^§^ | | | |
| No | 255 | 18.0 (6.0) | reference |
| Yes | 154 | 20.0 (6.5) | **2.0 (0.5 to 3.5)^*^** |
| Fetus sex^¶^ | | | |
| Male and female | 13 | 18.0 (8.0) | reference |
| Male or both male | 222 | 18.5 (6.0) | 0.5 (-3.5 to 5.0) |
| Female or both female | 171 | 19.0 (6.5) | 1.0 (-3.5 to 5.0) |
| Intention to induce abortion | | | |
| No | 350 | 18.5 (6.0) | reference |
| Yes | 59 | 21.0 (7.0) | **2.5 (1.0 to 4.0)^*^** |
| Being less than 28 weeks pregnant | | | |
| No | 361 | 18.5 (6.0) | reference |
| Yes | 48 | 20.5 (7.0) | **2.0 (0.1 to 4.0)^*^** |
| Abnormal results in the initial pregnancy tests^¥^ | | | |
| No | 234 | 18.0 (6.5) | reference |
| Yes | 175 | 19.5 (6.0) | **1.5 (0.1 to 2.5)^*^** |
| Perceived Social Support (assessed using MSPSS-12)^€^ | | | |
| High | 226 | 17.0 (6.5) | reference |
| Moderate | 178 | 20.5 (6.0) | **3.5 (2.0 to 5.0)^***^** |
| Low | 5 | 30.0 (5.5) | **13.0 (6.0 to 20.0)**^*^**^**^** |
| Lack of standard antenatal visits (at least four visits) | | | |
| No | 388 | 18.5 (6.0) | reference |
| Yes | 21 | 19.5 (8.0) | 1.0 (-2.0 to 4.0) |
| Experience of violence against women during the current pregnancy^£^; | | | |
| Psychological violence |  |  |  |
| No | 155 | 16.0 (5.5) | reference |
| Yes | 254 | 20.5 (6.0) | **4.5 (3.5 to 6.0)^***^** |
| Physical violence | | | |
| No | 328 | 18.0 (6.0) | reference |
| Yes | 81 | 22.5 (6.5) | **4.5 (3.0 to 6.0)^***^** |
| Sexual violence | | | |
| No | 356 | 18.0 (6.0) | reference |
| Yes | 53 | 22.0 (7.0) | **4.0 (2.0 to 6.0)^***^** |
| Any violence | | | |
| No | 138 | 15.5 (5.5) | reference |
| yes | 271 | 20.5 (6.0) | **5.0 (3.5 to 6.0)^***^** |
| History of hospitalization during the current pregnancy | | | |
| No | 340 | 18.0 (6.0) | reference |
| Yes | 69 | 20.0 (7.0) | **2.0 (0.5 to 3.5) ^*^** |
| Average sleep less or more than 8-9 hours during the day or night | | | |
| Yes | 137 | 17.5 (6.0) | reference |
| No | 272 | 19.5(6.5) | **2.0 (0.5 to 3.5)^*^** |
| Dissatisfaction with sleep quality during the past month | | | |
| No | 349 | 18.5 (6.0) | reference |
| Yes | 60 | 21.0 (7.0) | **2.5 (1.0 to 4.0)^**^** |

* P < 0.05, ** P < 0.005, *** P < 0.001

† Measured by perceived stress scale (PSS-10) with attainable range score 0-40; the higher score, the more stress

‡ All analysis were done using the unadjusted general linear model.

§ Including diabetes mellitus, hypertension, hypo/hyperthyroid, anemia, renal or cardiovascular diseases, infertility and others,

¶ Three cases were triplet pregnancy

¥ Those who had at least one abnormal result in their initial pregnancy laboratory tests such as TSH (Thyroid Stimulating Hormone), FBS (Fast Blood Sugar), CBC (Complete Blood Count), urine analysis and others.

€ Measured by multidimensional scale of perceived social support (MSPSS-12) with range score of 1-5, 1.0-2.33 low support, 2.34-3.67 moderate support, 3.68-5.0 high support,

£ Measured by WHO violence against women (VAW-13), experiences considered as “yes” when women have reported once or sometimes or often for at least one of the relevant items.
